# Three-dimensional characteristics analysis of maglev train with rotating operation conditions

**DOI:** 10.1371/journal.pone.0361708

**Published:** 2026-11-05

**Authors:** Huaimiao Zhu, Yuxin Guo, Bo Gao, Yaohua Ma, Baolong Yu, Zhixuan Zhang, Lei Zhang, Mingyuan Hu

**Affiliations:** 1 School of Electrical Engineering, Shandong Huayu University of Technology, Dezhou, China; 2 School of Mechanical and Electrical Engineering, Jiujiang Polytechnic University of Science and Technology, Jiujiang, China; 3 State Grid Shanxi Electric Power Research InstituteTaiyuan, China; 4 School of Electrical Engineering, Beijing Jiaotong University, Beijing, China; Tongji University, CHINA

## Abstract

Traction, suspension and guidance are characteristics of core electromagnetic performance of high-speed maglev. When the vehicle passes through curves or uneven ground, it is prone to generating significant three-dimensional (3-D) force fluctuations, which have a significant impact on the safety and stability of the maglev. This paper will discuss the effects of transient motion states on the suspension force, traction force and guidance force of maglev. First, general topology and 3-D distribution of electromagnetic forces of system is described. Then, force expressions in 3-D state are developed analytically taking into account the body translation and rotation. Lastly, the variation of force during the roll, pitch and lateral deviation under conditions are tested. The relevant conclusions indicate that the multi-degree-of-freedom motion of the vehicle will change the fluctuation values of 3-D electromagnetic forces, necessitating a magnetic levitation power device with a relatively large electromagnetic stiffness value, the design of a magnetic levitation power device requires a relatively large electromagnetic stiffness value.

## 1. Introduction

In order to implement the commercial operation of the electro-dynamic suspension superconducting maglev train, the system must be inexpensive and of a minimal maintenance requirement especially in terms of the minimization of costs of the guideway combination coils [1]. The single set of electromagnetic windings could be used as one way to reduce the cost of ground coils because this device can deliver traction, suspension and guidance at the same time [2–4]. The principles of control, operation and analysis of this high-speed maglev have already been presented above, and the fundamental electromagnetic properties have been pictorially demonstrated by use of some numerical models [4–9].

Moreover, controlling the magnetic field from the superconducting coils is a key task. By applying magnetic shielding to reduce flux leakage, the volume of shielding material can be kept to a minimum. An optimized strategy of magnetic shielding was suggested by Takashi Sasakawa and it was able to work both experimentally and numerically [10]. Subsequently, another superconducting professional suggested a newer initial process of design of magnetic shielding and tested its functionality in a magnetically levitated train [11]. Naoto Tagawa worked on the minimization of the magnetic field in the vehicle to less than 0.5 mT through the careful choice of shielding material and the design of the shielding structure according to the various vehicle parts [12]. Hiroshi Yoshioka looked at the configuration of superconducting coils in maglev systems with the aim of minimizing the weight of shielding material by demonstrating that shorter coils in the ends of the superconducting assemblies can be used effectively to reduce the weight of the shielding material [13, 14].

With the benefits of having combination coils on the track and superconducting structures on the train, the current maglev power system topology is defined. The combination coils in their guideway are positioned asymmetrical between the upper and lower coils, which are useful in reducing the take-off velocity and decreasing the maintenance price [15]. The sizes of the superconducting coils are also unequal and this helps in suppressing leakage flux as well as reduces the mass of shielding that is needed [16–19].

The various operating conditions on the three-dimensional (3-D) electromagnetic forces are then explored. The characteristics of maglev topology and 3-D forces are presented first. Second, an analytical calculation (AC) is used to analyze 3-D electromagnetic forces when coupling with multi axes of attitude, and the analytical equations are established. Finally, the 3-D electromagnetic force characteristics are evaluated through finite element analysis and analytical calculation, based on the coil parameters of the EDS high-speed maglev. The findings will be an effective source of information on the dynamics of transient 3-D forces when the maglev train rides in various motion states.

## 2. Topology Structure and Force Characteristics Analysis of the Maglev

### 2.1. The topology structure

Fig 1 shows the high-speed maglev layout. The track coil, through its inverting series design, achieves a net coupled magnetic flux of zero at the equilibrium position, significantly enhancing the levitation-resistance ratio. This includes the “8”-shaped zero-flux coil structure installed on the side walls, which can integrate both levitation and guidance functions. and Fig 2 illustrates the power system. Unequal superconducting coils are mounted on each side of the bogie, while asymmetric combination coils are installed on the guideway sidewalls. The train’s coil set has shorter coils at the ends and longer coils at the center. Track coils are connected via cable feeders, with each unit having asymmetric top and bottom windings in reverse at the mid-point. Front and rear coils are connected through ground coils and powered via a three-phase guideway connection.

**Fig 1:**
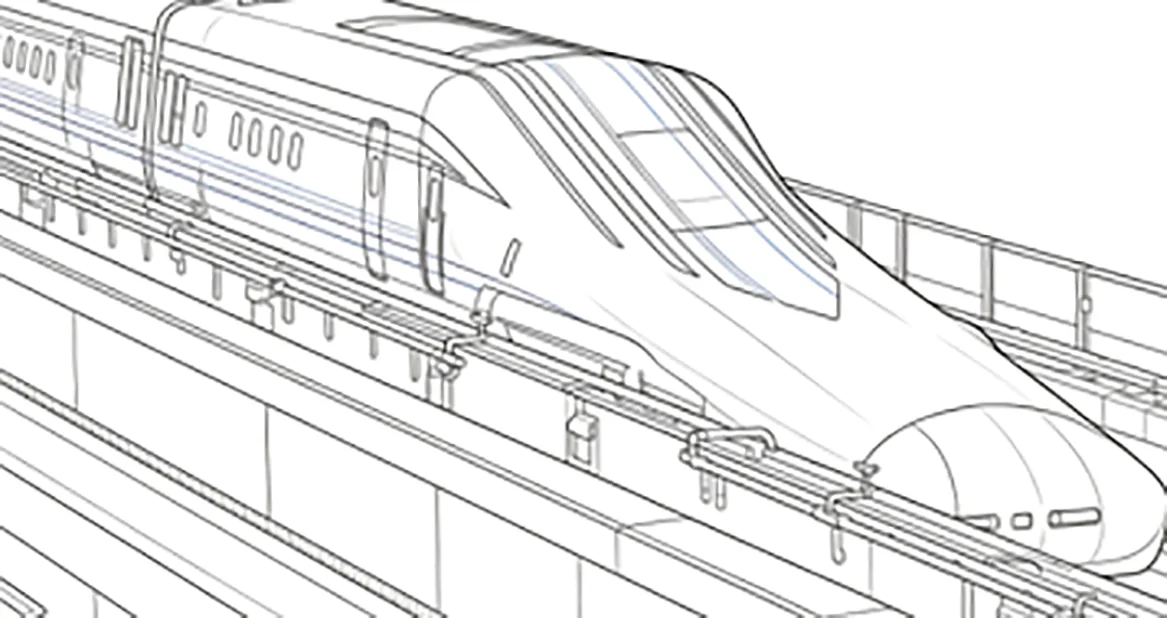
The structure diagram of EDS high-speed maglev train.

**Fig 2:**
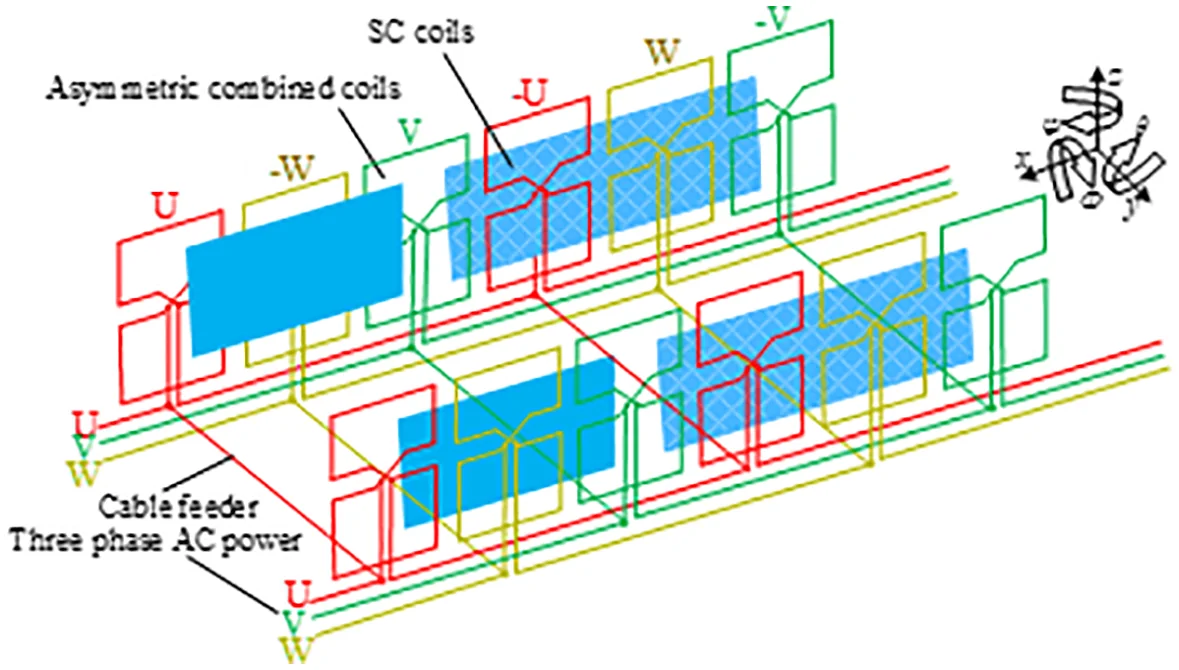
The power structure diagram of EDS high-speed maglev train (with scroll- ϕ, pitch- θ and deviation- ψ attitude).

### 2.2. The 3-D characteristics

Fig 3 shows the traction, suspension, and guidance force characteristics of the maglev. The three-dimensional electromagnetic force behavior under various angular degrees of freedom is described as follows. In the case of scroll attitude: when the bogie has counterclockwise torque, the side nearest to the guideway produces greater suspension and guidance force than the side farther away. With the pitch attitude: under clockwise pitch torque, the suspension and magnetic drag forces of the superconducting coils at the front increase, while those at the rear decrease. In the case of deviation attitude: under clockwise deviation, the guidance and magnetic drag forces at the front and rear experience significant variations. Additionally, the magnetic flux and air gap changes caused by these three attitudes affect the traction force of the maglev.

**Fig 3:**
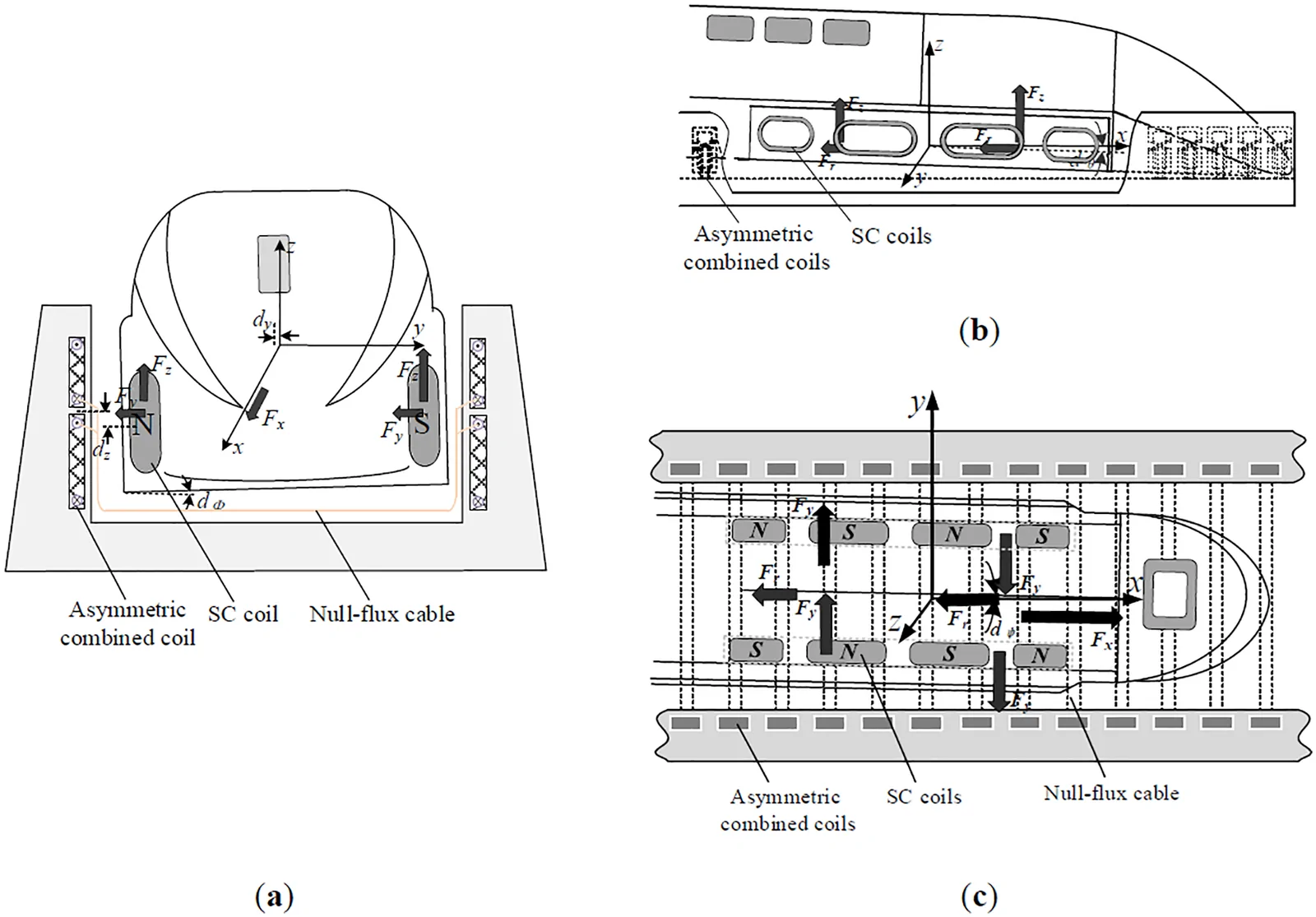
Diagrammatic illustration of 3-D force characteristics of the maglev (arrow length indicates peak force): (a) 3-D scroll electromagnetic force. (**b**) 3-D electromagnetic force under pitch condition. (c) 3-D electromagnetic force under deviation condition.

## 3. The 3-D Force Calculation of The Maglev

The system is assumed to experience a vertical deviation ($d_{z}$), lateral offset ($d_{y}$), scroll counter clockwise ($d_{\phi }$), pitch clockwise ($d_{\theta }$) and deviation clockwise ($d_{\psi }$) of the maglev, as shown in Fig 3. The track winding set is arranged asymmetrically, made up of four coils on the top and bottom sections, differing in size intrinsic properties and assuming that the induced electromotive force (EMF) of each coil is $\vec{e_{UL}},\vec{e_{BL}},\vec{e_{UR}},\vec{e_{BR}}$. Where, $\vec{e_{UL}}$: EMF in the upper-left rectangular coils; $\vec{e_{BL}}$: EMF in the below-left rectangular coils; $\vec{e_{UR}}$: EMF in the upper-right rectangular coils; $\vec{e_{BR}}$: EMF that appears in the lower-right element of the coil set, and the EMF equations are built following Lenz’s governing law of induction:


$$\begin{array}{c} \vec{e_{UR}}=-N_{iU}\frac{d\phi_{U}}{dt}=jwN_{iU}\left\{\phi_{U}+\frac{\partial \phi_{U}}{\partial z}(d_{z}+d_{zR}-d_{zP})+\frac{\partial \phi_{U}}{\partial y}(d_{y}+d_{yY})-\frac{\partial \phi_{U}}{\partial x}d_{xY}\right\}, \end{array}$$ (1)

$$\begin{array}{c} \vec{e_{BR}}=-N_{iB}\frac{d\phi_{B}}{dt}=jwN_{iB}\left\{\phi_{B}+\frac{\partial \phi_{B}}{\partial z}(d_{z}+d_{zR}-d_{zP})+\frac{\partial \phi_{B}}{\partial y}(d_{y}+d_{yY})-\frac{\partial \phi_{B}}{\partial x}d_{xY}\right\}, \end{array}$$ (2)

$$\begin{array}{c} \vec{e_{UL}}=-N_{iU}\frac{d\phi_{U}}{dt}=jwN_{iU}\left\{\phi_{U}+\frac{\partial \phi_{U}}{\partial z}(d_{z}-d_{zR}-d_{zP})-\frac{\partial \phi_{U}}{\partial y}(d_{y}+d_{yY})+\frac{\partial \phi_{U}}{\partial x}d_{xY}\right\}, \end{array}$$ (3)

$$\begin{array}{c} \vec{e_{BL}}=-N_{iB}\frac{d\phi_{B}}{dt}=jwN_{iB}\left\{\phi_{B}+\frac{\partial \phi_{B}}{\partial z}(d_{z}-d_{zR}-d_{zP})-\frac{\partial \phi_{B}}{\partial y}(d_{y}+d_{yY})+\frac{\partial \phi_{B}}{\partial x}d_{xY}\right\}, \end{array}$$ (4)

$$\begin{array}{c} \phi_{i}=\sum_{k=-\infty }^{+\infty }P_{i}\left(m\right)(-1{)}^{l-1}exp(-ja_{k}x_{0l})\;(i=U,B), \end{array}$$ (5)

$$\begin{array}{c} P_{i}=\sum_{n=1}^{+\infty }\frac{16\mu_{\text{0}}n_{sl}I_{sl}}{L_{sl}W_{sl}}k_{l}f_{\text{0}}\left(k,n\right)f_{1i}\left(k,n\right)exp(-k_{l}y_{d})cos\left[a_{n}\left(d_{z}\pm Z_{pi}\right)\right]\;(i=U,B), \end{array}$$ (6)

$$\begin{array}{c} d_{zR}=y_{\text{0}}d_{\phi }\;d_{zP}=x_{0l}d_{\theta }\;d_{yY}=x_{0l}d_{\psi }\;d_{xY}=y_{\text{0}}d_{\psi }, \end{array}$$ (7)

$$\begin{array}{c} a_{k}=k\pi /L_{sl}\;a_{n}=n\pi /W_{sl}\;k_{l}=\sqrt{{a_{k}}^{\text{2}}+{a_{n}}^{\text{2}}}\;, \end{array}$$ (8)

Where, $x=n_{n}l_{c}-vt$, *n*_*n*_ is the number of track coils, $y_{d}$ is the length of air gap, $y_{\text{0}}$ is the transverse distance between bogie center and superconducting coils center, $n_{sl}$ is the turns of superconducting coils, $I_{sl}$ is the current of superconducting coils, $x_{0l}$ express the positions in the advancing direction of each superconducting coil, $Z_{pi}$ is the distance between upper and lower center of track coils, the function $f_{\text{0}}$ and $f_{1i}$ is the shape factor of superconducting coils and combination coils, respectively; $N_{iU}$ and $N_{iB}$ is the turn number of upper and lower track coils, respectively; *k* and *n* is the Fourier decomposition coefficient in *x*-direction and *z*-direction, respectively.

The mathematical forms for the suspension, guidance, and traction currents are given as follows:


$$\begin{array}{c} \vec{i_{L}}=\sum_{k=-\infty }^{+\infty }\frac{jkw}{Z_{\text{1}}}(P_{\text{1}}+\frac{\partial P_{\text{1}}}{\partial z}d_{z}-\frac{\partial P_{\text{1}}}{\partial \theta }d_{\theta })exp\left(ja_{k}x\right), \end{array}$$ (9)

$$\begin{array}{c} \vec{i_{GU}}=\sum_{k=-\infty }^{+\infty }-\vec{i_{G1}}+\frac{{Z_{B}}^{\prime }}{Z_{\text{1}}}\vec{i_{G2}},\text{\textasciitilde{}}\vec{i_{GB}}=\sum_{k=-\infty }^{+\infty }\vec{i_{G1}}+\frac{{Z_{U}}^{\prime }}{Z_{\text{1}}}\vec{i_{G2}}, \end{array}$$ (10)

$$\begin{array}{c} \vec{i_{PU}}=\sum_{k=-\infty }^{+\infty }\frac{{Z_{B}}^{\prime }}{Z_{\text{1}}}\vec{i_{P}},\text{\textasciitilde{}}\vec{i_{PB}}=\sum_{k=-\infty }^{+\infty }\frac{{Z_{U}}^{\prime }}{Z_{\text{1}}}\vec{i_{P}}, \end{array}$$ (11)

$$\begin{array}{c} P_{\text{1}}=P_{B}-P_{U},\text{\textasciitilde{}}P_{\text{2}}={Z_{U}}^{\prime }P_{B}+{Z_{B}}^{\prime }P_{U} \end{array}$$ (12)

$$\begin{array}{c} \vec{i_{P}}=\frac{jkwP_{\text{2}}exp\left(ja_{k}x\right)-Z_{\text{1}}e_{\text{0}}}{Z_{\text{2}}}, \end{array}$$ (13)

$$\begin{array}{c} \vec{i_{Gi}}=\sum_{k=-\infty }^{+\infty }\frac{jkw}{Z_{i}}\left(\frac{\partial P_{i}}{\partial y}dy+\frac{\partial P_{i}}{\partial \phi }d\phi +\frac{\partial P_{i}}{\partial \psi }d\psi \right)exp\left(ja_{k}x\right)\;, \end{array}$$ (14)

The suspension force (*F*_*z*_), guidance force (*F*_*y*_), traction force (*F*_*x*_) are obtained:

Suspension force produced by suspension current in track coils:


$$\begin{array}{c} F_{zL}=L_{p}Re\left[i_{L}\frac{d({\phi_{B}}^{*}-{\phi_{U}}^{*})}{dz}\right]=L_{p}\sum_{k=-\infty }^{\infty }\frac{jkw}{Z_{\text{1}}}(P_{\text{1}}P_{1z}+{P_{1z}}^{\text{2}}d_{z}-P_{1z}P_{1\theta }d_{\theta }),\text{\textasciitilde{}}P_{ij}=\partial P_{i}/\partial j\text{(}i=1,2\;j=\phi ,\theta ,\psi \text{)} \end{array}$$ (15)

Suspension force produced by traction current in track coils:


$$\begin{array}{c} F_{zP}=2L_{p}Re[i_{PU}\frac{d{\phi^{*}}_{U}}{dz}+i_{PB}\frac{d{\phi^{*}}_{B}}{dz}]=\frac{L_{p}i_{p}}{2{\left|Z_{\text{1}}\left(1\right)\right|}^{\text{2}}}\left[Im\left({Z_{\text{1}}}^{*}\left(1\right)P_{2z}\left(1\right)\right)cos\left(wt)-Re({Z_{\text{1}}}^{*}\left(1\right)P_{2z}\left(1\right)\right)sin\left(wt\right)\right], \end{array}$$ (16)

Guidance force produced by guidance current in track coils:


$$\begin{array}{c} F_{yG}=L_{p}Re[i_{GU}\frac{d{\phi^{*}}_{U}}{dy}+i_{GB}\frac{d{\phi^{*}}_{B}}{dy}]=L_{p}\sum_{k=-\infty }^{\infty }\frac{jkw}{Z_{\text{1}}}(P_{\text{1}}P_{1y}+\frac{{P_{2y}}^{\text{2}}}{Z_{\text{2}}}dy), \end{array}$$ (17)

Guidance force generated by air gap length difference in track coils:


$$\begin{array}{c} F_{yg}=L_{p}Re\left[i_{L}\frac{d{\phi^{*}}_{B}-{\phi^{*}}_{U}}{dY}\right]=L_{p}\sum_{k=-\infty }^{\infty }\frac{jkw}{Z_{\text{1}}}(P_{\text{1}}P_{1Y}+P_{1z}P_{1Y}d_{z}-P_{1\theta }P_{1Y}d_{\theta }), \end{array}$$ (18)

Traction force generated by traction current in track coils:


$$\begin{array}{c} F_{x}=2L_{p}Re[i_{PU}\frac{d{\phi^{*}}_{U}}{dx}+i_{PB}\frac{d{\phi^{*}}_{B}}{dx}]=\frac{L_{p}i_{P}a_{k}\left(1\right)}{2{\left|Z_{\text{1}}\left(1\right)\right|}^{\text{2}}}\left[Re\left(Z_{\text{1}}\left(1\right)P_{\text{2}}\left(1\right)\right)cos\left(wt)+Im(Z_{\text{1}}\left(1\right)P_{\text{2}}\left(1\right)\right)sin\left(wt\right)\right], \end{array}$$ (19)

A comprehensive nomenclature Table 1 is shown below.

**Table 1:** The symbols, units, and their physical meanings of above formulas.

Symbol	Unit	Physical Meaning
$e_{ul}$	V	EMF in the upper-left rectangular coil
$e_{bl}$	V	EMF in the below-left rectangular coil
$e_{ur}$	V	EMF in the upper-right rectangular coil
$e_{br}$	V	EMF in the lower-right rectangular coil
*n*_*n*_	–	Number of track coils
δ	m	Length of the air gap
y	m	Transverse distance between bogie center and superconducting coils center
$N_{s}$	–	Turns of superconducting coils
$I_{s}$	A	Current of superconducting coils
$x_{i}$	m	Position in the advancing direction of each superconducting coil
d	m	Distance between upper and lower center of track coils
*Nu*	–	Turn number of upper track coils
$N_{l}$	–	Turn number of lower track coils
k	1/m	Fourier decomposition coefficient in the 𝑥-direction
n	1/m	Fourier decomposition coefficient in the 𝑧-direction
$i_{z}$	A	Suspension current
$i_{y}$	A	Guidance current
$i_{x}$	A	Traction current
$F_{z}$	N	Suspension force
$F_{y}$	N	Guidance force
$F_{x}$	N	Traction force

## 4. The 3-D Force Analysis by AC and FEA of The Maglev

To examine how the maglev’s forces behave in three dimensions under different operating conditions, the structural and circuit parameters of the suspension and propulsion system used in the high-speed maglev were applied, as listed in Table 2 and Table 3 [20–22]. The resulting 3-D force characteristics were computed and then compared with simulations carried out in ANSYS 19.2. The comparison covers both the total electromagnetic force and the changes observed in its three-axis components.

**Table 2:** The structure parameters of one maglev power structure.

Track coils	Value	Superconducting coils	Value
Length (m)	10.8	Length (m)	5.4
Width (m)	0.76	Width (m)	0.50
Number of coils	48	Number of coils	8
Polar distance (m)	0.45	Polar distance (m)	1.05/1.5
Upper coil width (m)	0.235	Turns	1400-2000
Lower coil width (m)	0.375	Length (m)	5.4
Turns	24	Width (m)	0.50

**Table 3:** The circuit parameters of one maglev power structure.

Track coils	Value	Superconducting coils	Value
Upper resistance (*R*_*U*_/ mΩ)	12.23	Resistance	0
Lower resistance (*R*_*B*_/ mΩ)	12.93	Temperature (*K*)	<20
Upper-inductance (*L*_*U*_/mH)	0.24	Operation current (*I*_sl_/A)	500
Lower-inductance (*L*_*B*_/mH)	0.36	Sync-speed (m/s)	135
Traction current (A)	390	Power angle (deg.)	40
Frequency (*f*/Hz)	50	Nominal voltage(kV)	0
Upper resistance (*R*_*U*_/ mΩ)	12.23		

In finite element model of the maglev, the secondary windings adopt NbTi superconducting material with direct current, which is magnetic part; the primary windings are made of aluminum wire material, which is non-magnetic part. The primary winding is equipped with three-phase sinusoidal alternating current provided by an external power supply. Further, the primary windings are set as stationary part, the secondary windings can follow the bogie to move with multiple degrees of freedom in 3-D space. The 3-D meth type adopts tetrahedral mesh (Tetra/Mixed), the 3-D mesh generation algorithm adopts robust (octree) method, the number of nodes and mesh are 253508 and 140837, the computation hardware uses: CPU (64 cores, 128 threads, 2.9G main frequency), Memory (DDR4 RECC 64G), Hard disk (Samsung solidity 256G) and Graphics card (onboard integrated VGA interface).

The analytical calculation model has the advantages of wider range of solutions, simpler formulation, shorter computation times and satisfactory precision. The FEA method takes 48–72 hours, while the analytical modeling only takes about 2 minutes.

### 4.1. Three-Dimensional Electromagnetic Force of the Maglev

Fig 4 shows how the forces of 3-D electromagnetism of the maglev change with the rotating angle. Fig 4(a) gives the behavior of suspension force over an angle of varying scroll and pitch. When the scroll angle becomes larger, the suspension force is incrementally raised but when the pitch angle becomes larger, the suspension force decreases. The guidance force behavior at various scroll and deviation angles is represented in Fig 4(b). The increase in guidance force with the scroll angle is accompanied by a rise-then-fall trend with deviation angle. The magnetic drag force at different scroll and deviation angles is presented in Fig 4(c); the scroll and deviation angles also led to slow decrease in the peak magnetic resistance. Lastly, Fig 4(d) shows the traction force variation when scroll, pitch and deviation angles are changed. Variations in these angles produce small effects on the maximum traction force which increases gradually with increasing excitation magnetomotive force.

**Fig 4:**
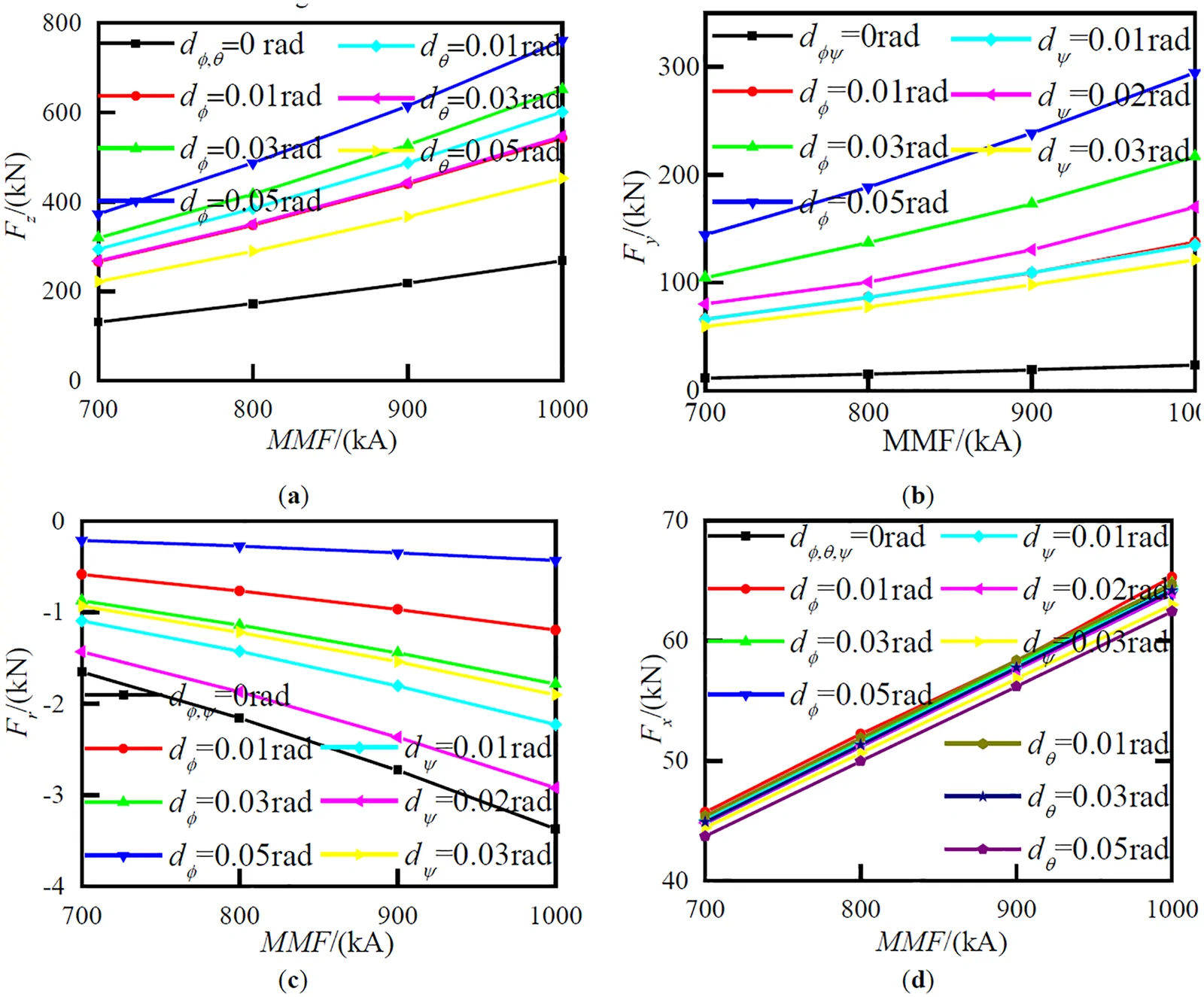
Curves of variation of 3-D electromagnetic forces at various scroll, pitch and deviation angle: (a) Suspension force in scroll and pitch. (**b**) Scroll conditions and deviation conditions variation of guidance force. (**c**) The variation of magnetic drag force under scroll and deviation conditions. (**d**) Scroll, pitch, and deviation variation of traction force.

### 4.2. Variations in Three-dimensional Electromagnetic Force

Fig 5 indicates that 3-D electromagnetic forces of the maglev change when the rotational angle of the magnet changes. Fig 5(a) demonstrates the variation of the suspension force when the scroll and pitch angle is varied. It rises first in the direction of the scroll and pitch angles and falls. Fig 5(b) shows the variation in guidance force at different scroll and deviation angles. Although fluctuations increase first and at the end with scroll angle, it decreases slowly with increasing deviation angle. The variations of magnetic drag force at scroll, pitch and deviation angle are presented in Fig 5(c). Peaks of the fluctuation go up and down, first in scroll angle, and then in steady increasing amounts in pitch and deviation angles. The variation in traction force, (Fig 5(d)). There is minimal sensitivity of traction force variation with the change in scroll or pitch but the variation is high with the increase in deviation angle.

**Fig 5:**
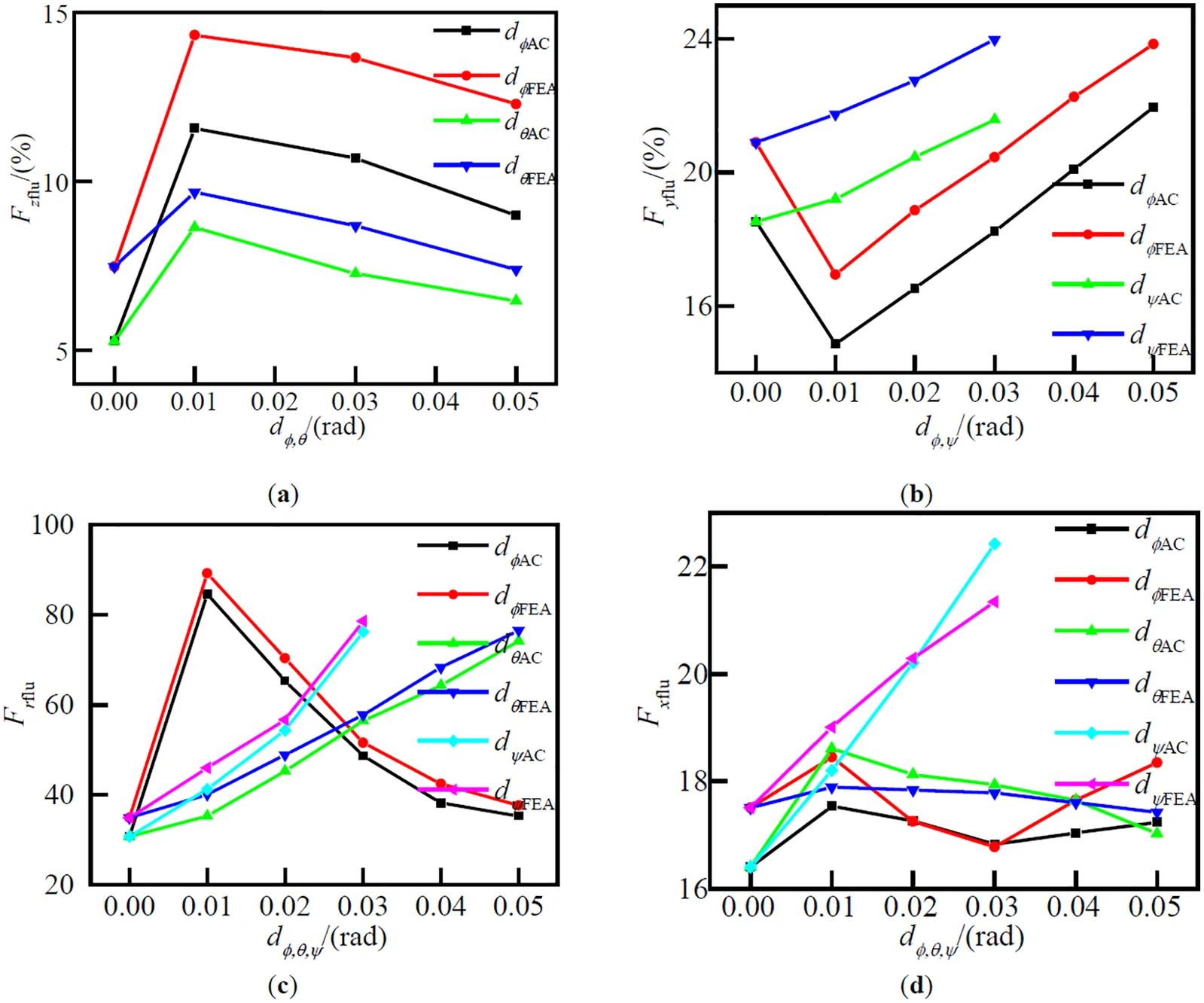
Flutter curves of 3-D electromagnetic forces with variation in the conditions of operation: (a) The fluctuation of suspension force in the cases of scroll and pitch. (**b**) Scroll and deviation conditions of guidance force variation. (**c**) Fluctuation of magnetic drag force at scroll, pitch, and deviation angles. (**d**) Fluctuation in traction force at scroll, deviation and pitch conditions.

## 5. Conclusions

In the manuscript, the influence of various transient operating conditions of maglev train on 3-D electromagnetic force characteristics of the maglev is analyzed. Firstly, the 3-D power model of the maglev under various train attitude (including translation and rotation) is established, and the 3-D electromagnetic force calculation expressions of the maglev are obtained. Then, when the train is in different transient operation attitude, the variation law of 3-D electromagnetic force characteristics is analyzed by analytical method and finite element method. And the following main conclusions are obtained: (1) The increase of train scroll angle leads to the increase of suspension force and guiding force; (2) The increase of train pitch angle leads to the increase of suspension-to-drag force ratio; (3) The increase of train deviation angle leads to the increase of traction force and force fluctuation. This integrated magnetic levitation system faces issues such as increased electromagnetic force fluctuations, coil stress concentration and temperature rise, which require further optimization in the future.
